# Zinc, Iron and Vitamins A, C and E Are Associated with Obesity, Inflammation, Lipid Profile and Insulin Resistance in Mexican School-Aged Children

**DOI:** 10.3390/nu5125012

**Published:** 2013-12-10

**Authors:** Olga Patricia García, Dolores Ronquillo, María del Carmen Caamaño, Guadalupe Martínez, Mariela Camacho, Viridiana López, Jorge L. Rosado

**Affiliations:** 1 School of Natural Sciences, Autonomous University of Queretaro, Av. De la Ciencia S/N, Juriquilla, Querétaro 76230, Mexico; E-Mails: lolitaron@hotmail.com (D.R.); mccaamano@hotmail.com (M.C.C.); nutgua@hotmail.com (G.M.); marielacamachob@yahoo.com.mx (M.C.); lilian_viridiana@hotmail.com (V.L.); jlrosado@prodigy.net.mx (J.L.R.); 2 Research and Development Center for Chronic Diseases (Cindetec) A.C., Jurica 122, Parque Industrial Querétaro, Querétaro 76220, Mexico

**Keywords:** children, micronutrients, obesity

## Abstract

The objective of this cross-sectional study was to evaluate the relationship between micronutrient status and obesity, lipids, insulin resistance and chronic inflammation in children. Weight, height, waist circumference and body composition (dual-energy X-ray absorptiometry (DEXA)) were determined in 197 school-aged children. Lipids, glucose, insulin, C-reactive protein (CRP), zinc, iron and vitamins A, C and E were analyzed in blood. Vitamin C and vitamin E:lipids were negatively associated with Body Mass Index (BMI), waist-to-height ratio (WHR) and body and abdominal fat (*p* < 0.05). Vitamin A was positively associated with BMI, BMI-for-age, WHR and abdominal fat (*p* < 0.05). Iron and vitamin E:lipids were negatively associated with insulin (*p* < 0.05). Vitamins A, C and E and iron were negatively associated with CRP (*p* < 0.05). Interaction analysis showed that children who were overweight and obese who also had low concentrations of vitamin A had higher CRP and lower triglycerides (*p* < 0.1), children with low vitamin E had significantly lower glucose and triglycerides (*p* < 0.1) and higher low-density lipoprotein (LDL) concentrations (*p* < 0.05), and children with low zinc concentrations had higher insulin resistance compared with children with adequate weight (*p* < 0.05). In conclusion, low vitamin C concentration and vitamin E:lipids were associated with obesity. Furthermore, low concentrations of zinc, vitamins A and E in children who were overweight and obese were associated with lipids, inflammation and insulin resistance.

## 1. Introduction

In the past few years, the prevalence of childhood obesity in Mexico and other developing countries in the world has increased at alarming proportions. The combined prevalence of being overweight and obesity in school-aged Mexican children is 34.4% (19.8% and 14.6%, respectively). For girls, this combined prevalence is 32% and for boys is 36.9%. Childhood obesity increases the risk for certain health conditions, such as hypertension, cardiovascular disease, diabetes and metabolic syndrome. In a population of school-aged children in Mexico, with a high prevalence of being overweight and obesity (35%), the prevalence of high triglyceride concentration was 57%; 33% had elevated low-density lipoprotein (LDL). Only 6% had adequate high-density lipoprotein (HDL) concentrations, and 65% had high LDL-HDL ratios [1]. It has also been observed in school-aged Mexican children that obesity not only increases the risk of cardiovascular disease, but also chronic inflammation [2]. Preventive measures are needed to decrease the prevalence of childhood obesity in Mexico and to decrease the risk of metabolic syndrome in this and other populations, as well.

Recent studies suggest that micronutrient deficiencies may contribute to fat deposition and chronic inflammation [3,4,5]. A higher risk of low concentrations of iron, zinc, vitamin A, vitamin E and vitamin C have been observed in obese children and adolescents compared to children and adolescents with normal weight [6,7,8,9]*.* The deficiency of these micronutrients may increase the risk of developing obesity. Vitamins A, C and E, for example, have been shown to decrease or inhibit the expression of leptin, in both humans and animal models [4,10,11,12].

In Mexico, the prevalence of micronutrient deficiencies in school-aged children is high: approximately 16.6% of the children have anemia; 13% have iron deficiency; 23.6% have zinc deficiency; and 30% have vitamin C deficiency [13,14,15]. Thus, it is possible that the high prevalence of micronutrient deficiencies might be contributing to the development of obesity, particularly in rural areas, where the prevalence of micronutrient deficiencies is higher. In women from rural areas, concentrations of zinc and vitamin C were associated with obesity, adiposity and leptin concentrations [4]. Furthermore, low zinc concentrations were associated with chronic inflammation [5]. The relationship between micronutrient status with obesity and comorbidities, such as cardiovascular disease, chronic inflammation and risk of diabetes in school-aged children, is not known.

The objective of this study was to evaluate the relationship between micronutrient status and obesity, lipid profile, insulin resistance and low-grade systemic inflammation in school-aged children from a rural area in Queretaro, Mexico.

## 2. Materials and Methods

### 2.1. Subjects and Experimental Design

A total of 197 school-aged children (6–10.5 years) participated in a cross-sectional study. Children were recruited from two rural communities, Amazcala and San Rafael, in the State of Querétaro in Mexico. Parents received oral and written information about the study procedures. This study was conducted according to the guidelines of the Declaration of Helsinki, and all procedures involving human subjects were approved by the Human Research Committee of the School of Natural Sciences at the Universidad Autónoma de Querétaro (UAQ) (Project ID: FCN-2009-02). Written informed consent was obtained from all subjects’ parents or caretaker. Children that had consumed a micronutrient supplement for the past month, had type I diabetes or had any physical or mental disability were not included in the study.

The sample size was calculated considering an estimated micronutrient deficiency prevalence of 30%, an odds ratio of being obese for the deficient children relative to non-deficient children of 2.35, with a statistical power of 0.8 and a type I error of 0.05. A total of 180 children were needed.

Children whose parents signed consent forms were evaluated in their schools, and their weight and height were measured. Parents were interviewed to determine their socioeconomic status (SES). During the same visit, a single fasting blood sample was collected from the participants for the biochemical determinations.

On a separate day, children were transported with one of their parents or tutor from their communities to the Nutrition Clinic at UAQ for body composition determination, blood pressure measurement and dietary intake evaluation.

### 2.2. Anthropometry and Body Composition

Weight and height were measured in duplicate, non-consecutively, by trained personnel following World Health Organization procedures [16]. Children were weighed in light clothing, without sweaters or shoes, using an electronic scale (SECA Mod. 813, Hamburg, Germany) to the nearest 0.1 g. Height was determined using a portable stadiometer (SECA Mod 206, Hamburg, Germany) with 0.1-cm precision. A child was considered overweight with a Body Mass Index-for-age (BMI-for-age) from the World Health Organization growth charts above one standard deviation and obese above two standard deviations [17]. 

Whole body composition analysis to determine body fat percent, lean mass and fat-free mass was done using dual-energy X-ray absorptiometry (DEXA) (Hologic Mod Explorer, Bedford, MA, USA). Analysis was carried out by a certified technician. Abdominal fat mass and abdominal fat percent were estimated following a procedure previously described by Hill *et al.* [18]. Excess body fat for girls was considered above 30% and above 25% for boys [19,20]. 

### 2.3. Blood Pressure

Blood pressure was measured in duplicate with at least one hour’s difference between measurements, using a digital wrist blood pressure monitor (Citizen Mod CH606, Tokyo, Japan). High blood pressure was defined with a systolic or diastolic blood pressure higher than the 95th percentile [21].

### 2.4. Blood Analysis

A fasting blood sample was collected by venipuncture from each subject on the first visit to the community health clinic. Children and their parents were instructed not to eat anything at least 12 h before the blood sample was collected early in the morning. Plasma and serum were separated in blood samples by centrifugation at 1800–2000 rpm for 15 min, and aliquots were stored at −70 °C for later analysis. Blood analysis included vitamins A, C and E, total iron, zinc, glucose, insulin, total cholesterol, low-density lipoprotein (LDL), high-density lipoprotein (HDL), triglycerides and C-reactive protein (CRP). All laboratory analyses were performed in duplicate at the Human Nutrition Laboratory of UAQ.

Vitamins A and E were measured in serum using the modified reported technique by Bieri *et al.* [22]. These vitamins were measured simultaneously by reverse phase high pressure liquid chromatography (HPLC) (Mod 2996, Waters Associates, Milford, MA, USA), at a wavelength of 300 nm, using the corresponding U.S. Pharmacopeial (USP) certified retinol and alpha-tocopherol standards, with a C18 column (Waters, New Braunfels, TX, USA) and a mobile phase of 100% methanol (J.T.Baker, Deventer, Netherlands). Vitamin A deficiency was considered with retinol concentrations <10 μg/dL and low concentrations <20 μg/dL [23]. Vitamin E deficiency was defined with a concentration of alpha-tocopherol <3 μg/mL and low concentrations <5 μg/mL [24]. When using the vitamin E:lipids ratio, vitamin E deficiency was considered with <0.8 mg/g [25]. Serum vitamin C was determined by reverse phase HPLC, with a photodiode detector (Waters Mod 2996, Waters Associates, Milford, MA, USA), at a wavelength of 254 nm, using a C18 column (Waters, New Braunfels, TX, USA) and a mobile phase of NaH_2_PO_4_ 0.01 M and ethylenediaminetetraacetic acid (EDTA) 0.2 mM (J.T.Baker, Deventer, Netherlands), as previously reported [26]. Vitamin C deficiency was considered with concentrations of ascorbic acid <2 μg/mL and low concentrations with levels of *<*4 μg/mL [23].

Total iron concentration in serum was measured using a commercial kit (Iron Ferrozine, Elitech, Sées, France) and a spectrophotometer (Perkin Elmer, Mod Zeeman 5100). Low iron concentrations were considered <60 μg/dL and deficiency with concentrations <45 μg/dL [27]. Zinc concentrations were measured in serum by atomic absorption spectrometry (AAnalyst 7000, Perkin Elmer Instruments, Norwalk, CT, USA). Zinc deficiency was defined with zinc plasma concentrations <65 mg/L [28].

Plasma HDL cholesterol and LDL cholesterol were measured by spectrophotometry (Genesis 20 ThermoSpectronic, Thermo Electron Corp, Madison, WI, USA) using commercially available kits (Cholesterol HDL, Elitech, Sées, France; Cholesterol LDL, Spinreact, Sant Esteve de Bas, Spain). Triglycerides and total cholesterol were determined in plasma using commercially available kits (Cholesterol, Elitech, Sées, France; Triglycerides, Elitech, Sées, France) using a clinical chemical analyzer (Bayer RA-50, Bayer Diagnostics, Dublin, Ireland). High total cholesterol concentrations were defined as ≥200 mg/dL, high LDL with concentrations ≥130 mg/dL, high triglycerides with concentrations ≥100 mg/dL for children <9 years of age and ≥130 mg/dL for children >10 years of age and low HDL with concentrations <40 mg/dL [29].

Fasting glucose was measured in plasma by a colorimetric/enzymatic method using a commercial kit (Glucose Elitech, Sées, France) and a clinical analyzer (Bayer RA-50, Bayer Diagnostics, Leverkusen, Germany). Insulin concentration in serum was determined by a commercial ELISA kit (Bio Quant, San Diego, CA, USA) using a microplate photometer (Multiskan Ascent, Thermo Electron Corporation, Waltham, MA, USA). Insulin resistance was determined using the Homeostatic Model Assessment (HOMA) with the following formula: HOMA = (insulin × glucose)/22.5 [30]. A child with fasting glucose concentrations ≥100 mg/dL was considered pre-diabetic [31]. Insulin resistance was defined with a HOMA value >3.16 [30].

CRP was quantified in serum using a commercial high sensitivity ELISA kit (Bioquant, San Diego CA, USA) with a Multiskan Ascent microplate photometer (Thermo Electron Corporation, Waltham, MA, USA).

### 2.5. Socioeconomic Status

Socioeconomic status (SES) was evaluated using a validated questionnaire that has been used in previous studies [32,33] and included information regarding living conditions, belongings and crowding. The SES variables were included in the statistical analysis models. 

### 2.6. Diet Evaluation

Children’s habitual diet was evaluated by previously trained nutritionist using a quantitative food frequency questionnaire that was applied to the children and his/her mother at the same time. Daily nutrient intake was calculated using food composition tables from the United States Department of Agriculture (USDA) [34] and from the National Institute of Medical Sciences and Nutrition “Salvador Zubirán” [35].

### 2.7. Physical Activity

Physical activity was measured using a previously validated questionnaire for school-aged children [36]. Mothers were asked the total hours of physical activity, type of activities and frequency of these activities performed by their children during the previous week. According to the number of the metabolic equivalent of task (Met), the activities were divided according to their intensity as light (<3 Mets), moderate (3–5 Mets) and vigorous (>6 Mets) [37].

### 2.8. Statistical Analysis

Descriptive analyses were performed for all variables. Dependent variables that did not meet a normal distribution were transformed to their natural logarithm to perform the statistical tests that require this assumption, and geometric means are presented in transformed variables. Pearson correlations between micronutrient variables and outcome variables (anthropometry, body fat and biochemical markers) were carried out. In addition, linear models were performed to predict biochemical markers from a low concentration of micronutrients and the interaction of each micronutrient with the presence of being overweight and obesity, adjusted for age, crowding and father’s education in years. The variables, insulin and HOMA Index, could not achieve the normal distribution; thus, a generalized linear model that uses the gamma distribution with the log link function was used to evaluate the same associations. Statistical significance was set at a probability level of <0.05 and <0.1 for interactions. All data was analyzed using SPSS version 19 (IBM^®^ SPSS^®^, Armonk, NY, USA).

## 3. Results

Complete questionnaires and laboratory data were obtained from 197 children. General characteristics of the population are described in Table 1. Approximately 18% of the children had high triglycerides concentrations. Seven point one percent had a low concentration of HDL, and the combined prevalence of being overweight and obesity was high (44%).

**Table 1:** General characteristics of the children that participated in the study according to their Body Mass Index (BMI) (*n* = 197). HOMA, Homeostatic Model Assessment.

Characteristics	Means	SD ^1^
*Demography*		
Age, months	99	16.6
Crowding ^2^, ppr ^3^	2.94	1.57
Father’s education, years	7.15	2.82
Mother’s education, years	6.67	2.58
*Anthropometry*		
Weight, kg	29.49	1.30
Height, m	128.06	8.85
BMI ^2^, kg/cm^2^	18.07	1.18
BMI for age, *Z*-score	0.99	1.21
Waist ^2^, cm	63.88	1.15
Waist to height ratio ^2^	50.00	1.11
*Body Composition*		
Body fat, %	31.13	6.40
Abdominal fat ^2^, kg	436.76	1.73
*Clinical and biochemical measures*		
Systolic ^2^, mmHg	91.15	1.11
Diastolic ^2^, mmHg	63.34	1.11
C-reactive protein ^2^, mg/dL	1.62	2.03
Glucose, mg/dL	68.23	6.89
Triglycerides ^2^, mg/dL	87.56	1.56
Total cholesterol, mg/dL	128.75	29.50
Low density lipoproteins, mg/dL	77.18	19.74
High density lipoproteins, mg/dL	47.08	10.35
Insulin ^2^, µIU/mL	3.39	1.47
HOMA ^2^	0.57	1.50
*Micronutrient concentrations*		
Vitamin A, µg/dL	29.72	7.20
Vitamin C, µg/mL	4.25	1.51
Vitamin E, µg/mL	5.80	1.42
Vitamin E:lipids ratio mg/g	2.68	0.68
Iron, µg/dL	87.78	29.07
Zinc, µg/dL	74.38	13.29
*Physical activity*		
Vigorous, h/day	3.11	4.03
Moderate, h/day	11.93	8.73
Light, h/day	48.62	11.22

^1^ SD, standard deviation; ^2^ values are geometric means; ^3^ ppr, people per room.

The prevalence of low concentrations of vitamin E, vitamin C and iron and the prevalence of zinc deficiency were high in this population (Table 2). Vitamin A or vitamin E deficiencies were very low.

**Table 2:** Prevalence of low concentrations and deficiencies of zinc, iron, vitamin A, vitamin C and vitamin E in the children that participated in the study (*n* = 197).

Micronutrient status	%
Vitamin A < 20 µg/dL	7.1
Vitamin C < 4 µg/mL	38.1
Vitamin C < 2 µg/mL	8.1
Vitamin E < 5 µg/mL	32.5
Vitamin E < 3 µg/mL	2.0
Vitamin E:lipids < 0.8 mg/g	0.0
Iron < 60 µg/dL	17.9
Iron < 45 µg/dL	6.6
Zinc < 65 µg/dL	24.9

Dietary intake, including micronutrient intake, is shown in Table 3. The distribution of energy intake from carbohydrate, protein and fat is adequate in this population. Intake of fiber and all the micronutrients studied was below the recommended intake. The main sources of cereals and legumes consumed among this population were maize tortillas (154 g/day) and beans (76 g/day), respectively. Fruit drinks (naturally and artificially flavored) (112 g/day) and sodas (179 g/day) are highly consumed (>380 g/day), while fruit and vegetable intake is low (74 g of fruits and 40 g of vegetables per day). Furthermore, the daily intake of foods from animal origin, such as milk and meat, is low, only 12 g/day and 65 g/day, respectively.

Adjusted correlations show a negative and significant association between vitamin C concentration and waist/height ratio, body fat and abdominal fat (*p* < 0.05) (Table 4). On the contrary, vitamin A concentration was positively associated with BMI, BMI-for-age, waist circumference, waist/height ratio and abdominal fat (*p* < 0.05). Vitamin E concentration was also positively associated with waist/height ratio (*p* < 0.05). When adjusting for lipids, vitamin E concentrations were negatively associated with all the measures of obesity (*p* < 0.05). Low concentrations of iron and vitamins A, C and E were associated with higher CRP concentrations (*p* < 0.05). High insulin concentrations and insulin resistance were associated with low iron and vitamin E concentrations and the vitamin E:lipids ratio (*p* < 0.05). Finally, concentrations of total cholesterol, triglycerides and LDL were significantly and positively associated with vitamin A, vitamin E and zinc and negatively associated with vitamin E:lipids (*p* < 0.05).

**Table 3:** .Energy, macronutrients and zinc, iron, vitamins A and C intake of the children that participated in the study (*n* = 197).

Nutrient intake	Means	95% CI
Energy (kcal) ^1^	1529.70	1472.2, 1588.7
Carbohydrates (g) ^1,2^	228.8	225.1, 232.7
% Energy from carbohydrates	59.4	58.5, 60.2
Protein (g) ^1,2^	48.7	47.4, 49.9
% Energy from protein	12.8	12.4, 13.1
Fat (g) ^1,2^	47.0	45.6, 48.4
% Energy from fat	27.9	27.1, 28.7
Fiber (g) ^1,2^	12.8	12.2, 13.5
Calcium (mg) ^1,2^	707.0	682.1, 732.8
Iron (mg) ^1,2^	10.7	10.4, 11.1
Zinc (mg) ^2^	5.1	3.5, 6.8
Vitamin A (µg) ^1,2^	419.5	390.0, 451.0
Vitamin C (mg) ^1,2^	44.1	40.0, 48.6
Vitamin E (mg) ^1,2^	2.8	2.6, 3.0
*Nutrient intake below RI* ^3^		
Calcium	75.1%	
Iron	77.2%	
Zinc	92.9%	
Vitamin A	52.8%	
Vitamin C	29.9%	
Vitamin E	98.0%	

^1^ GM, geometric means and 95% confidence interval; ^2^ estimated values are adjusted for energy; ^3^ recommended intakes for children aged four to eight years and nine to 13 years are as follows: calcium < 800 mg and < 1200 mg, iron < 13 mg and <17 mg, zinc < 6.6 mg and <11.6 mg, vitamin A < 400 µg and <580 µg, vitamin C < 25 mg and <45 mg, vitamin E < 7 mg and <11 mg, respectively.

**Figure 1:**
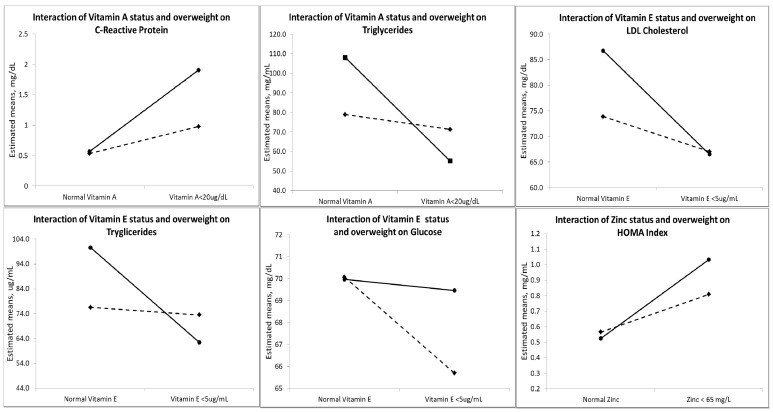
Interaction between being overweight and obesity and micronutrients on C-reactive protein, triglycerides, low-density lipoprotein, glucose and insulin resistance in overweight/obese and normal weight children (*n* = 197). Continuous lines represent children with a BMI *Z*-score > 1 SD, and the dotted lines represent children with a BMI *Z*-score ≤ 1 SD.

Interaction analysis showed that children who were overweight and obese that had low concentrations of vitamin A had significantly higher CRP concentrations (*p* < 0.05) and lower triglycerides (*p* < 0.1). Children with low vitamin E had significantly lower glucose (*p* < 0.1) and triglycerides (*p* < 0.05) and higher LDL concentrations (*p* < 0.05), and children with low zinc concentrations had higher insulin concentration and insulin resistance compared with children with adequate weight with low concentrations of these micronutrients (*p* < 0.05) (Table 5, Figure 1).

**Table 4:** Adjusted correlations between micronutrients and anthropometry, body composition and biochemical variables in school-aged children from rural Mexico (*n* = 197) ^1^.

Variables	Vitamin A (µg/dL)	Vitamin E (µg/mL)	Vitamin E:Lipids ratio	Vitamin C (µg/mL)	Iron (µg/dL)	Zinc (μg/dL)
BMI, Kg/cm^2^	0.223 *	0.115	−0.426 *	−0.116	−0.137	0.028
BMI for age, Zscore	0.219 *	0.100	−0.423 *	−0.077	−0.119	0.044
Waist, cm	0.178 *	0.116	−0.435 *	−0.142	−0.128	0.048
Waist to height ratio	0.178 *	0.080 *	−0.443 *	−0.157 *	−0.114	0.03
Body fat, %	0.116	0.081	−0.441 *	−0.231 *	−0.092	−0.026
Abdominal fat, Kg	0.192 *	0.137	−0.413 *	−0.204 *	−0.111	0.004
CRP, mg/dL ^2^	−0.248 *	−0.188 *	0.096	−0.143 *	−0.285 *	−0.085
Glucose, mg/dL	0.011	0.050	0.029	0.141	−0.153 *	0.031
Triglycerides, mg/dL	0.332 *	0.428 *	−0.542 *	0.030	0.023	0.118
Total cholesterol, mg/dL	0.340 *	0.500 *	−0.449 *	0.041	0.090	0.224 *
HDL, mg/dL ^2^	0.125	0.247 *	0.215 *	0.150 *	0.184 *	0.166 *
LDL, mg/dL ^2^	0.309 *	0.438 *	−0.441 *	−0.006	0.05	0.208 *
Insulin, μIU/mL	−0.070	−0.245 *	−0.322 *	0.007	−0.150 *	0.034
HOMA Index	−0.076	−0.233 *	−0.313 *	0.024	−0.171 *	0.028

^1^ Values are correlation coefficients adjusted for crowding and children’s age; ^2^ CRP, C-reactive protein; HDL, high density lipoproteins; LDL, low density lipoprotein; * significance level: <0.05.

**Table 5:** Linear models to predict biochemical variables from micronutrients and their interaction with being overweight and obesity (*n* = 197) ^1^.

Independent variables	C-Reactive Protein, mg/dL		Glucose, mg/dL		Triglycerides, mg/dL		Total cholesterol, mg/dL		Low density lipoprotein, mg/dL		High density lipoproteins, mg/dL		Insulin, μIU/mL		HOMA Index	
	Beta ^1^ 95% CI	*p* value	Beta 95% CI	*p* value	Beta 95% CI	*p* value	Beta 95% CI	*p* value	Beta 95% CI	*p* value	Beta 95% CI	*p* value	Beta 95% CI	*p* value	Beta 95% CI	*p* value
BMI Zscore > 1 SD	0.77 −0.06, 1.61	0.069	2.75 −5.90, 11.40	0.531	−24.02 −72.32, 24.29	0.328	−6.07 −39.42, 27.27	0.720	−4.70 −30.15, 20.74	0.715	0.06 −12.05, 12.16	0.993	0.70 0.24, 1.16	0.003	0.78 0.30, 1.26	0.001
Iron < 60 µg/dL	0.25 −0.09, 0.58	0.149	0.48 −2.99, 3.95	0.784	−10.30 −29.69, 9.08	0.296	−4.59 −17.95, 8.78	0.499	−8.49 −19.01, 2.03	0.113	0.03 −4.83, 4.89	0.990	0.01 −0.17, 0.19	0.922	0.03 −0.16, 0.22	0.783
Iron < 60 µg/mL * BMI *Z*-score > 1 SD		0.489		0.319		0.765		0.577		0.299		0.627		0.501		0.355
Zinc < 65 μg/dL	−0.15 −0.50, 0.21	0.418	0.81 −2.90, 4.51	0.668	11.50 −9.26, 32.25	0.276	−1.95 −16.23, 12.33	0.788	4.58 −6.63, 15.79	0.420	−3.52 −8.73, 1.68	0.183	0.23 0.03, 0.43	0.027	0.23 0.02, 0.44	0.032
Zinc < 65 μg/dL * BMI *Z*-score > 1 SD		0.667		0.377		0.223		0.562		0.184		0.811		0.020		0.045
Vitamin A < 20 µg/dL	1.34 0.64, 2.04	0.000	1.41 −5.86, 8.69	0.702	−52.86 −93.48, −12.23	0.011	−17.95 −45.99, 10.08	0.208	−4.14 −27.09, 18.82	0.722	1.71 −8.48, 11.89	0.741	0.22 −0.17, 0.60	0.274	0.30 −0.11, 0.71	0.147
Vitamin A < 20 µg/dL * BMI *Z*-score > 1 SD		0.037		0.580		0.068		0.167		0.781		0.760		0.424		0.260
Vitamin E < 5 µg/mL	0.10 −0.21, 0.41	0.516	−0.51 −3.74, 2.71	0.755	−38.13 −56.14, −20.11	0.000	−23.91 −36.34, −11.48	0.000	−20.23 −29.54, −10.91	0.000	−3.90 −8.42, 0.62	0.090	0.16 −0.01, 0.33	0.066	0.15 −0.03, 0.33	0.106
Vitamin E < 5 µg/mL * BMI *Z*-score > 1 SD		0.840		0.096		0.007		0.273		0.044		0.650		0.739		0.457
Vitamin C < 4 µg/mL	0.16 −0.11, 0.44	0.240	−1.98 −4.83, 0.87	0.172	12.68 −3.22, 28.58	0.117	2.32 −8.65, 13.29	0.677	1.10 −7.22, 9.42	0.795	−2.15 −6.13, 1.84	0.290	0.004 −0.15, 0.155	0.957	−0.03 −0.19, 0.13	0.734
Vitamin C < 4 µg/mL * BMI *Z*-score > 1 SD		0.171		0.788		0.604		0.973		0.267		0.434		0.894		0.888

^1^ Values are beta coefficients from linear models adjusted for age, crowding and father’s education in years. Since the dependent variables of these models could not achieve the normal distribution, these models were performed with the gamma distribution.

## 4. Discussion

In the present study, zinc, iron and vitamin A, C and E status relate differently to measures of obesity, inflammation, lipid concentrations and insulin resistance in school-aged children from rural areas in Mexico.

A low vitamin C concentration was associated with higher body fat, abdominal fat and waist/height ratio. Similar results have been shown in adult populations with regards to being overweight and obesity, where the concentration of vitamin C was inversely associated with BMI, body fat and waist circumference [4,38]. Vitamin C may reduce adiposity through a number of different mechanisms. Ascorbic acid has been shown to modulate adipocyte lipolysis [39,40], inhibit inflammatory response [41] and inhibit leptin concentration [10]. Supplementing rats with vitamin C reduced the circulating levels of leptin and decreased body weight and adiposity in a rat model [42]. Similarly to vitamin C, vitamin E concentrations adjusted by lipids were inversely associated with all markers of obesity. Lower vitamin E concentrations have also been associated with obesity in some populations [43,44] and, as with vitamin C, this association could be due to its role in leptin metabolism [12,45,46].

In this population, vitamin A concentration was positively associated with measures of obesity. These results differ from Viroonudomphol *et al*. (2003), who found that low concentrations of vitamin A in Thai adults who were overweight and obese were associated with higher weight, BMI and hip circumference [47]. In a study in women from the same rural areas in Mexico, vitamin A was positively related to measures of obesity, but only on the lowest terciles of BMI (<30 kg/m^2^), waist circumference (<84.6 cm) and body fat (<36%) [48]. The effect of vitamin A on adipogenesis, specifically retinoic acid, appears to be dose-dependent, suggesting that the relationship between vitamin A and adiposity differs in individuals with higher BMI and body fat content [4].

Vitamins A and E concentrations were positively related to triglycerides and total cholesterol concentration. Our results agree with those of Obeid *et al.* [49], where higher vitamin A and E concentrations were associated with higher concentrations of total cholesterol, LDL and triglycerides in a Lebanese adult population. The retinol-binding protein:serum retinol ratio has also been associated with high lipid concentration, particularly triglycerides, in children in Switzerland [6]. These results may be explained by the roles that vitamins A and E have on lipid metabolism. For example, vitamin A coordinates lipid metabolism through the retinaldehyde dehydrogenase 1 enzyme [50]. Vitamin E is involved in lipids metabolism, mainly by protecting the lipids from oxidation and preventing the formation of oxidative damage [51]. Thus, higher lipid concentrations will require higher vitamin A and vitamin E concentrations, and deficiencies of these vitamins may increase the risk of cardiovascular disease starting at an early age. Given the importance of these vitamins in lipids metabolism, public health strategies should focus on the impact of vitamin A and E deficiencies on an abnormal lipid profile.

C-reactive protein has been associated with obesity in both adults and children [52,53,54] and with an increase of cardiovascular risk factors and metabolic syndrome [55,56]. In the population studied, concentrations of vitamin A, vitamin C, vitamin E and iron were negatively associated with concentrations of CRP. In addition, children that were overweight and obese and had low concentrations of vitamin A had higher concentrations of CRP compared with normal weight children. Our results are similar to those reported in other populations [52,57,58]. Richardson *et al.* [52] found lower iron concentrations in children with high risk CRP values of >3 mg/L compared to children with low CRP values. Low levels of vitamin C have been associated with higher CRP concentrations in dialysis patients [59], in men with impaired vascular endothelial function [57] and in an adult population in Japan [60]. Similarly, an inverse relationship was found between vitamin A and vitamin E with CRP in both children and adult populations [54,58,61], and supplementing with vitamin E has been shown to reduce CRP levels and oxidative stress [62,63]. The role vitamin E has in inflammation and oxidative stress may ameliorate the positive relationship observed in this study between vitamin E and lipid profile. Thus, these micronutrients might have anti-inflammatory properties and may play an important role in the prevention of chronic inflammation associated with obesity in the early stages of life.

Vitamin E, iron and zinc concentrations were related with glucose and insulin in the population studied. Children with normal weight had significantly lower concentrations of glucose when they had vitamin E deficiency compared with overweight and obese children. In contrast, animal models have demonstrated that vitamin E participates in glucose metabolism and may be a protective factor against type 2 diabetes [64]. High insulin concentrations and the HOMA Index were related to low iron concentrations*.* It has been observed in studies in human and animal models that iron is involved in the regulation of insulin and glucose [65,66]. Iron overload, for example, has been associated with an increased risk of developing type 2 diabetes through the metabolism of adiponectin [67,68]. Furthermore, a higher HOMA index was observed in zinc-deficient children who were overweight and obese than in children without zinc deficiency. These results agree with those found by Ortega *et al.* [69], who observed an increased risk of insulin resistance with age, higher BMI and low concentrations of zinc in school-aged children. Zinc is known to play an important role in insulin metabolism, specifically in its synthesis, storage and secretion [70]. Furthermore, the expression of the adipocytokine zinc-α (2) glycoprotein (ZAG), involved in the stimulation of lipolysis in the adipocyte, is reduced in obesity and has also been related with higher insulin resistance [71]. These results suggest that zinc deficiency may be a potential risk factor for insulin resistance and type 2 diabetes in the later stages of life.

One major limitation of the present study is that, since it is a cross-sectional study, causality cannot be established. Furthermore, with the exception of zinc, the prevalence of micronutrient deficiencies was low, even when the intake of all micronutrients was below the recommended intake for school-aged children. The relationship between obesity and micronutrients could be different in obese populations with a higher prevalence of micronutrient deficiencies.

## 5. Conclusions

In conclusion, low vitamin C concentration and the vitamin E:lipids ratio were associated with obesity. In addition, low concentrations of vitamins A and E and zinc in children who were overweight and obese were associated with lipids, inflammation and insulin resistance. More studies are needed to explore the causality of these relationships. Furthermore, future research should focus on the effectiveness of providing micronutrients to prevent obesity and its comorbidities in this age group.
